# Development and validation of the socially shared regulated learning questionnaire: insights from a second-order confirmatory factor analysis

**DOI:** 10.3389/fpsyg.2025.1635325

**Published:** 2025-09-08

**Authors:** Yongkang Yang, Zhenni He, Yinuo Wei, Erlong Tang

**Affiliations:** ^1^School of Education, City University of Macau, Macao, Macao SAR, China; ^2^School of Artificial Intelligence, Nanning Normal University, Nanning, China; ^3^Department of English and German Studies, Autonomous University of Barcelona, Bellaterra, Spain; ^4^School of Foreign Languages, Anqing Normal University, Anqing, China

**Keywords:** socially shared regulated learning, questionnaire validation, confirmatory factor analysis, collaborative learning, metacognition

## Abstract

This study addresses a critical methodological gap by developing and validating a psychometrically robust, multidimensional socially shared regulated learning (SSRL) questionnaire. Grounded in contemporary theories of collaborative learning and regulation, the questionnaire comprises four theoretically informed dimensions: Shared Goal Setting and Planning, Shared Monitoring and Control, Shared Reflection and Evaluation, and Shared Motivational and Emotional Regulation. Employing a two-phase methodological design, the study first refined questionnaire items through a think-aloud protocol (TAP) with a diverse student sample. Subsequently, the refined instrument was administered to 378 university students, and its structural validity and reliability were evaluated via second-order confirmatory factor analysis (CFA). Results demonstrated excellent internal consistency (*ω* ≥ 0.82 for all dimensions), robust model fit (CFI = 0.951; RMSEA = 0.050; SRMR = 0.038), and strong hierarchical relationships between dimensions and the overarching SSRL construct. This validated questionnaire provides educators and researchers with an effective, scalable tool for assessing collaborative regulation processes, thus offering insights into enhancing collaborative learning outcomes in diverse educational contexts.

## 1 Introduction

Learning is not an isolated process. After extensive theoretical and empirical development, scholars in contemporary educational psychology have shifted their focus. They now explore not only individual cognitive processes but also group dynamics and the mechanisms underlying social interaction and collaboration. Social constructivism stands as one of the key cornerstones of this paradigm shift. Vygotsky (1978) argued that learners’ understanding and construction of knowledge do not occur in isolation. Instead, they develop gradually through interactions with others, including linguistic exchanges and cultural transmissions. This well-established theory has provided a robust theoretical foundation for both researchers and learners to comprehend and engage in collaborative learning, profoundly influencing the evolution of subsequent, more specific theoretical frameworks.

Under the influence of this theory, traditional self-regulated learning (SRL) theory has gradually expanded to encompass collaborative learning contexts. SRL emphasizes that learners proactively set goals, monitor their learning processes, evaluate learning outcomes, and adjust their strategies accordingly to enhance both learning efficiency and quality (Zimmerman, 2000). Some extended theories further incorporate metacognitive skills and motivational factors (Pintrich, 2000) or examine the reciprocal interaction with the learning environment (Schunk and Zimmerman, 1998). Grounded in traditional SRL theory, Hadwin et al. (2011) notably proposed a perspective that highlights social interaction and collaborative regulation as central to learning processes, rather than mere extensions of individual self-regulation. This conceptualization was later elaborated within SSRL, as detailed in Panadero and Järvelä (2015), who argued that this approach better captures the complexity and multi-level nature of learning regulation in team-based collaboration.

The concept of SSRL emphasizes that, in group-based interactions, individuals co-construct goals and standards, monitor progress, provide feedback, and adjust strategies to foster shared cognitive regulation (Hadwin and Oshige, 2011). Traditionally, SSRL data have been collected by recording learners’ verbal behaviors, observing both verbal and nonverbal interactions, and assessing individual contributions and overall outcomes; yet in large-scale or otherwise constrained research contexts, questionnaire surveys offer an efficient means of rapidly gathering extensive evaluative data on students’ SSRL capacities. Despite broad theoretical consensus on SSRL’s importance, empirical investigations have been impeded by the lack of a rigorously validated, multidimensional measurement instrument and by the predominant use of single-phase surveys underpinned by minimal qualitative data-constraints that undermine both the interpretive depth of quantitative analyses and the generalizability of findings across diverse educational contexts.

To address these gaps, this study synthesizes and distills empirically validated SSRL frameworks proposed by various scholars and developed and validated a 24-item SSRL instrument comprising four dimensions—Shared Goal Setting and Planning, Shared Monitoring and Control, Shared Reflection and Evaluation, and Shared Motivational and Emotional Regulation. Employing a two-phase design, the first phase refines item wording via a think-aloud protocol (TAP) with a diverse student sample. The second phase administered the revised instrument to 378 undergraduates and assess its structural validity and reliability through confirmatory factor analysis (CFA). This study offers researchers and practitioners a psychometrically robust tool for assessing group-level regulatory processes, thereby advancing understanding of how socially shared regulation influences collaborative learning outcomes.

## 2 Literature review

### 2.1 Self-regulated learning (SRL)

SRL denotes learners’ active, constructive regulation of cognition, motivation, and behavior toward personally set goals. In SRL, students are active participants in metacognitive, motivational, and behavioral processes. They plan strategies aligned with their goals, monitor progress, and reflect on outcomes (Zimmerman, 1989; Pintrich, 2000). Pioneering models of SRL emphasize these processes. For example, Zimmerman’s social-cognitive model (1989) conceptualized SRL as a cyclical triadic interplay among personal, behavioral, and environmental factors. Winne and Hadwin (1998) proposed an information-processing model known as COPES, which stands for Conditions, Operations, Products, Evaluations, and Standards. This model outlines phases such as task definition, goal setting, study tactics, and adaptations, and emphasizes metacognitive monitoring at each stage. Boekaerts’ dual-path model (Boekaerts and Cascallar, 2006) posits that learners dynamically shift between a mastery-oriented (growth) pathway and a coping-oriented (wellbeing) pathway based on task appraisal. Subsequent work has extended these theories to incorporate affect and motivation. For instance, models of motivational regulation treat students’ shifting motivation as integral to SRL (Schwinger and Stiensmeier-Pelster, 2012), and others highlight students’ regulation of emotions and values as part of SRL. Moreover, contemporary research on SRL is increasingly situated in technology-rich learning. A recent systematic review (Lan and Zhou, 2025) finds that AI-based tools (e.g., adaptive chatbots, feedback systems) can scaffold students’ goal-setting, monitoring, and reflection phases—though they also raise concerns about maintaining learner autonomy. Together, these models and trends portray SRL as a complex, context-sensitive process. Importantly, most classic SRL theories focus on the individual learner. In practice, however, students’ self-regulatory processes are shaped by social and contextual influences. For example, Järvelä and Hadwin (2013) distinguish self-regulated learning (SRL, each student regulating alone) from co-regulated learning (CoRL, interactions triggering mutual regulation) and socially shared regulated learning (SSRL, fully collaborative group regulation). This framing highlights CoRL as an intermediate stage bridging solitary SRL and group-based SSRL.

### 2.2 Co-regulated learning (CoRL)

Co-regulated learning (CoRL) denotes interpersonal regulatory processes whereby learners’ planning, monitoring, and evaluation are guided, modeled, or prompted by others and by mediating artifacts, with regulatory control shifting across people and over time (McCaslin, 2009; Hadwin and Oshige, 2011; Järvelä and Hadwin, 2013). Rather than an individual property, CoRL is situated in social interaction: teachers, peers, and tools externalize goals and criteria, surface misunderstandings, and provide contingent feedback that helps learners appropriate effective regulatory routines. In classroom practice, this occurs in formative feedback, rubric-guided revision, or peer tutoring, where interlocutors articulate expectations, check progress, and adjust strategies in light of shared evidence (Andrade et al., 2021). Contemporary accounts treat such co-regulatory episodes as recurrent features of instruction and collaboration, and they are analytically distinct from self-regulated learning (SRL) at the individual level and from socially shared regulation of learning (SSRL) at the group level, yet dynamically related to both.

#### 2.2.1 CoRL as a transitional mechanism from SRL to SSRL

Within this study’s conceptual frame, CoRL functions as a linking mechanism between individually driven SRL and group-level SSRL. Through recurrent co-regulatory episodes, externally scaffolded moves, including goal articulation, strategy selection, progress checking, and evaluative judgment, are progressively internalized by individuals and, in collaborative settings, become mutually negotiated and jointly maintained. When regulatory intentions, standards, and monitoring routines are no longer located in a single actor but are co-constructed and sustained by the group, regulation attains the socially shared form captured by the SSRL construct (Järvelä and Hadwin, 2013; Allal, 2019). Rather than measuring CoRL in the present instrument, we theorize it as a proximal antecedent that catalyzes the coordinated forms of SSRL. Specifically, in this study SSRL is operationalized as four interrelated dimensions—shared planning and goal setting, shared monitoring and control, shared reflection and evaluation, and shared motivational and emotional regulation—which are elaborated in 2.3.3. This positioning clarifies the pathway from individual regulation to group-level sharing while preserving the construct boundaries required for valid measurement.

### 2.3 Socially shared regulation of learning (SSRL)

#### 2.3.1 Concept and theoretical foundations

While SRL has been extensively studied in individual contexts, its application in collaborative environments still has limitations. Within the continuous development of educational psychology, the concept of SSRL has emerged as a significant extension and deepening of SRL, particularly in contexts where joint learning is characterized by collaborative groups work (Panadero and Järvelä, 2015).

The theoretical foundation of SSRL builds on Vygotskian theories of social constructivism, which posits that learning and cognitive development are fundamentally social processes (Vygotsky, 1978). According to this perspective, higher-order mental functions are initially mediated through interaction with others before being internalized by the individual (Vygotsky, 2012). This social nature of learning has inspired an expansion of traditional views of SRL, shifting from purely individual processes to more collaborative forms of regulation (Schunk and Zimmerman, 2011).

In contrast, SSRL occurs when group members jointly regulate their collective learning processes. It most referred to group members through shared outcomes way to construct synthesize strategies, such as planning, goal setting, monitoring, evaluation (Hadwin et al., 2011). Unlike SRL, which focuses on individual cognitive, motivational, emotional aspects of learning, and CoRL which involves one learner guiding another’s regulation. SSRL extends these processes to the group level (Panadero, 2017), it emphasizes group members equal participation and shared control of the regulatory process (Rogat and Linnenbrink-Garcia, 2011).

#### 2.3.2 Questionnaires for measuring SSRL

SSRL encompasses multidimensional processes that enable groups to navigate the complexities of collaborative learning. However, understanding the multilevel regulatory activities involved in joint problem-solving and regulation remains inherently complex and variable, as these processes fluctuate dynamically across self-, co-, and shared-regulation modes (Vauras et al., 2003).

While growing recognition of SSRL’s educational value, the development of measurement tools (like questionnaires) according to SSRL frameworks remains challenging. Existing studies have identified some dimensions of SSRL, such as Lee (2014) identified group cooperation process to planning, goal setting, task monitoring, content monitoring, task evaluation, and content evaluation. Miller and Hadwin (2015) designed a regulation macro-script that breaks down collaborative tasks into three main phases and five key steps: (a) planning (Steps 1–3), (b) monitoring and evaluating (Steps 3 and 4), and (c) reflecting for adapting (Step 5). Li et al. (2022) empirically validated the role of planning and goal setting, task and content monitoring, and task and content evaluation in enhancing computational thinking. Sharma et al. (2024) think socially shared regulatory activities can involve planning, monitoring, controlling and reflecting on a group’s learning processes. O’Connell et al. (2023) identified four regulate factors in interdisciplinary groups: (a) goal setting and planning, (b) implementation, monitoring, and evaluation, (c) the role of supervisors, and (d) the impact of disciplines. Despite these advancements, current tools focus narrowly on cognitive and metacognitive dimensions, neglecting motivational, emotional aspects (Järvelä et al., 2015; Urban and Urban, 2025). Therefore, more work should be performed to develop comprehensive tools for test and validate the SSRL processes among student groups.

#### 2.3.3 Four dimensions involved in SSRL

Panadero and Järvelä (2015) proposed three design principles for supporting SSRL are introduced: (a) increasing learner awareness of their own and others’ learning processes, (b) supporting externalization of one’s own and others’ learning process and helping to share and interact, and (c) prompting acquisition and activation of regulatory processes. These principles align with and extend the empirically identified dimensions of SSRL. Building on these foundations of dimensions and principles, four dimensions have been identified: shared planning and goal setting, shared monitoring and control, shared reflection and evaluation, shared motivational and emotional regulation.

Effective SSRL begins with the co-construction of shared strategic plans and goals setting. Li et al. (2022) demonstrated that groups engaging in collaborative goal setting and planning significantly outperformed control groups in computational thinking tasks. This aligns with Hadwin et al. (2017) conceptualization of SSRL as a process where members set common goals together, share responsibility for strategy formulation, and coordinate adjustments to optimize problem-solving. In interdisciplinary project-based learning, Feng et al. (2025) found that shared planning fostered a unified understanding of task requirements and leveraged group strengths, though variations in disciplinary perspectives occasionally necessitated iterative goal negotiation. These findings indicated that shared goal setting is not static but evolves through dynamic interactions, balancing individual contributions with collective objectives (Järvenoja et al., 2013). Thus, shared planning and goal setting serves as the initial phase of SSRL and forms the foundation of its regulatory process.

Shared monitoring fuels collaborative learning in groups (Dindar et al., 2019), and provides support for students’ SSRL (Quackenbush and Bol, 2020). During task execution, groups engage in continuous monitoring of their progress and make necessary adjustments to their strategies. This dynamic process ensures that the group remains aligned with its goals and can adapt to emerging challenges. According to Sobocinski et al. (2020), the essence of shared monitoring lies in identifying key regulatory moments, which can be categorized into three dimensions: the monitoring target, valence, and phase. These dimensions help pinpoint critical points during the collaborative process that require regulation. Multimodal data reveal that groups require more intensive shared monitoring and control (making adjustments based on monitoring feedback) during the task redefinition phase, as they renegotiate task understanding or role distribution (Sobocinski et al., 2020). Moreover, structured peer monitoring has been shown to enhance cognitive outcomes and perceived competence while reducing anxiety and boredom (Larsen et al., 2020). Positioned as a second-order dimension, shared monitoring and control reflects its temporal position following goal establishment and its functional role in maintaining regulatory throughout collaborative tasks.

Shared reflection and evaluation play a pivotal role in sustaining collaborative learning by enabling groups to critically assess their progress, problem-solving processes, and group dynamics. Lin (2018) found that implementing shared reflection and evaluation mechanisms can moderately reduce the free-rider effect and enhance the level of SSRL. This is because group awareness reveals members’ collaborative behaviors and helps regulate participation, while peer evaluation encourages accountability by assessing individual contributions. Other studies have highlighted that reflection content focusing on critical and contextual aspects significantly improves the depth of collaborative reflection (Zheng et al., 2025), and content-evaluation is among the most frequently applied regulatory strategies during online collaborative learning (Shukor et al., 2015). To support the reflection phase of SSRL, students increasingly apply AI tools—such as simulators, exploratory learning environments, and interactive dashboards—as they provide data-driven feedback and promote metacognitive thinking (Kim et al., 2025). These tools enable groups to externalize their learning processes, assess outcomes, and identify areas for improvement. Therefore, shared reflection and evaluation not only involve retrospective analysis of group performance but also act as a forward-looking mechanism that enhances future collaboration through collective insight and informed decision-making.

Shared motivational and emotional regulation involves group members’ joint efforts to sustain engagement and manage affective states, maintaining a positive collaborative process. Research shows that when students hold different learning goals and expectations, motivational conflicts may arise (Järvelä et al., 2010). And difficulties in group communication can lead to socio-emotional challenges that weaken task focus (Barron, 2003). Vauras et al. (2003) linked motivation to shared regulatory efficacy, observing that high-ability peer groups with strong task orientation exhibited resilience in problem-solving. Rogat and Linnenbrink-Garcia (2011) found that groups with more positive socio-emotional interactions demonstrated higher-quality planning and monitoring. Effective groups often use motivational strategies such as praise, emphasizing task value, and stimulating focus to foster group engagement (Hogenkamp et al., 2021). Similarly, Törmänen et al. (2022) revealed that students with diverse emotional participation profiles contributed more actively to group regulation. However, motivational regulation in SSRL remains underexplored compared other dimensions, with Hadwin et al. (2011) calling for greater attention to how groups co-regulate motivation and emotional states. Therefore, this dimension is included to address one of a research gap and to clarify how motivational and emotional regulation supports effective SSRL.

In summary, these four dimensions provide a comprehensive framework for understanding how learners jointly regulate their cognition, behaviors, emotions, and goals in collaborative contexts. By synthesizing prior research on SSRL, this review not only deepens our understanding of SSRL mechanisms but also informs the theoretical basis for the development of the measurement tools employed in this study.

## 3 Methods

### 3.1 Participants

Students in Chinese higher education constituted the target population. In compulsory education, the Compulsory Education Curriculum Standards (2022 Edition) integrate group discussion and collaborative inquiry into classroom organization, but the policy framing positions teachers as the primary designers and managers of these activities, indicating comparatively stronger teacher orchestration than in universities (Ministry of Education of the People’s Republic of China, 2022). By contrast, since the launch of the “Golden Courses” initiative and the broader student-centered teaching reform, universities have increasingly emphasized inquiry-based, project-based, and collaborative learning, which reduces direct instruction and heightens the need for learner autonomy and co-regulation (Ministry of Education of the People’s Republic of China, 2018), making SSRL particularly salient in the tertiary context. We further delimited the age range to 18–35 to approximate the core undergraduate-to-doctoral population and to align with prevailing recruitment norms in China, where many entry-level academic posts explicitly set an upper age limit of 35, shaping doctoral timing and early career transitions (Horta and Li, 2024).

This study was conducted in two phases. In the pilot phase, six students from diverse academic disciplines and educational levels participated: two female doctoral candidates in education, one female master’s student in English, one male bachelor’s student in English, one male master’s student in computer science, and one male bachelor’s student in mathematics (age range = 19–26 years). They were recruited through a combination of faculty recommendations and convenience sampling at two universities. Eligibility criteria included (a) prior experience in at least one small-group learning activity or collaborative project and (b) willingness to participate in a 30–40-min online think-aloud protocol session conducted via Tencent Meeting.

In the subsequent main survey phase, data were collected via convenience sampling from undergraduate and graduate students at three public universities in Guangdong, Anhui, and Shanxi provinces. Of the 381 returned questionnaires, three were excluded due to age values outside the 18–35-year range, yielding a final sample of N = 378 (age range = 18–32 years, M = 20.09, SD = 2.80; 37.04% female, 62.96% male). At the beginning of the online questionnaire, all participants were presented with an information statement describing the purpose, procedures, and their rights in the study. Only those who selected “I agree to participate in this study” were able to proceed to the survey, thereby providing informed consent. The heterogeneity of the sample provided a robust empirical basis for testing the SSRL model. The study protocol was approved by the relevant institutional review board.

### 3.2 Instrumentation and procedure

#### 3.2.1 Questionnaire development (pilot study)

In the pilot phase, six students from different academic backgrounds were purposively sampled to complete a think-aloud protocol (TAP) via Tencent Meeting, evaluating the initial 24 items of the SSRL questionnaire. Each participant read every item aloud and verbalized any thoughts, confusions, or interpretations in real time. With participants’ consent, all sessions were audio- and video-recorded; each session lasted 30–40 min. After each round, the research team reviewed recordings to document pauses, questions, and suggestions, and revised the questionnaire accordingly (see Supplementary material 2).

The pilot study revealed four primary issues, which were addressed as follows: (a) ambiguous referent: participants questioned whether “group activities” extended beyond learning contexts, so the phrase was clarified as “small-group learning activities”; (b) subject confusion: items originally using “we” were revised to “I” to ensure individual reflection; (c) redundancy: semantically similar items prompted the addition of four reverse-scored items (2, 14, 18, and 24) to enhance discriminability and engagement; and (d) excessive length: long, complex sentences were simplified and rewritten for clarity.

#### 3.2.2 SSRL questionnaire

As shown in Supplementary material 1, the final SSRL questionnaire comprised 24 items rated on a seven-point Likert scale (1 = Never; 2 = Very rarely; 3 = Rarely; 4 = Occasionally; 5 = Sometimes; 6 = Often; 7 = Always) to enhance sensitivity and discriminability. Items 1–5 assessed Shared Goal Setting and Planning; items 6–10 assessed Shared Monitoring and Control; items 11–15 assessed Shared Reflection and Evaluation; and items 16–24 assessed Shared Motivational and Emotional Regulation. To mitigate response bias, items 2, 14, 18, and 24 were reverse-scored. In the present sample, the scale demonstrated excellent internal consistency (Cronbach’s *α* = 0.928). The revised SSRL questionnaire was administered in batches via Microsoft Forms to students at three public universities. Data (see Supplementary material 3) were exported to Excel and Python 3.12 for cleaning. Screening steps (range and consistency checks) were documented prior to analysis.

### 3.3 Data analysis

#### 3.3.1 Descriptive statistics

Items 2, 14, 18, and 24 were reverse-coded (reverse score = maximum + minimum − raw score). Item-level means, standard deviations, skewness, and kurtosis were computed in Python to assess univariate normality. Multivariate normality was evaluated with Mardia’s test to verify distributional assumptions for subsequent factor analyses. The cleaned dataset was saved in a plain-text format directly readable by Python 3.12 and Mplus 8.4 for subsequent analyses.

#### 3.3.2 Reliability analysis

Considering the unequal factor loadings among the scale items, which violates the *τ*-equivalence assumption of Cronbach’s α and may lead to systematic underestimation of reliability, McDonald’s *ω* was adopted as the primary indicator of internal consistency for both the overall SSRL scale and its subscales. For comparability with previous studies, Cronbach’s α was also computed for the overall scale, and its value is also reported in Section 4.2 for reviewers’ reference.

#### 3.3.3 Assessment of factorability

Prior to conducting confirmatory factor analysis, data factorability was evaluated in Python by computing the Kaiser–Meyer–Olkin (KMO) measure of sampling adequacy and Bartlett’s test of sphericity. Following established methodological guidelines, a KMO value of 0.60 or higher and a significant Bartlett’s test (*p* < 0.05) indicate that the correlation matrix contains sufficient common variance for factor extraction (Hair et al., 2019).

#### 3.3.4 Second-order confirmatory factor analysis

Because the four-dimensional framework of this study was *a priori* derived from an integrative synthesis of existing SSRL theory and corresponding scale dimensions, a second-order CFA was conducted directly, rather than performing exploratory factor analysis to test the hypothesized hierarchical structure. Given the departure from multivariate normality, the model was estimated in Mplus 8.4 using robust maximum likelihood (MLR), which provides robust standard errors and scaled test statistics (Hair et al., 2019). Model fit was assessed using the chi-square test (χ^2^), comparative fit index (CFI), Tucker–Lewis index (TLI), root mean square error of approximation (RMSEA), and standardized root mean square residual (SRMR). For RMSEA, we additionally report the p-close statistic, which tests the null hypothesis of close fit (RMSEA ≤ 0.05); a non-significant p-close (*p* > 0.05) indicates that close fit cannot be rejected (Kline, 2016). Consistent with widely accepted guidelines, values of CFI/TLI ≥ 0.90 and RMSEA/SRMR ≤ 0.08 indicate acceptable fit, whereas CFI ≥ 0.95 and RMSEA ≤ 0.06 reflect good fit (Hu and Bentler, 1999; Hair et al., 2019).

## 4 Results

### 4.1 Descriptive statistics

Descriptive-statistic ranges for the overall SSRL scale and its four subscales are displayed in Table 1. Item-level means fell between 3.63 and 4.77 (SD = 1.45–1.66), skewness between −0.74 and 0.48, and kurtosis between 2.47 and 3.27, indicating broadly acceptable univariate distributions. Mardia’s test of multivariate normality was significant for both skewness (b₁ = 188.78, *p* < 0.001) and kurtosis (b₂ = 1121.08, *p* < 0.001), suggesting that the multivariate normality assumption was not met.

**Table 1:** Descriptive-statistic ranges for overall SSRL and subscales.

Scale/subscale	M range	SD range	Skewness range	Kurtosis range
Overall SSRL	3.63–4.77	1.45–1.66	−0.74–0.48	2.47–3.27
Shared goal setting and planning	4.10–4.53	1.52–1.66	−0.62–0.23	2.47–2.81
Shared monitoring and control	4.28–4.45	1.47–1.54	−0.66– –0.47	2.85–3.06
Shared reflection and evaluation	3.83–4.50	1.51–1.55	−0.60–0.34	2.57–3.04
Shared motivational and emotional regulation	3.63–4.77	1.45–1.66	−0.74–0.48	2.59–3.27

### 4.2 Reliability analysis

McDonald’s *ω* for the overall SSRL scale was 0.960 (Cronbach’s *α* = 0.928), indicating excellent internal consistency. Subscale ω values were likewise strong, with Shared Goal Setting and Planning at 0.824, Shared Monitoring and Control at 0.935, Shared Reflection and Evaluation at 0.844, and Shared Motivational and Emotional Regulation at 0.888.

### 4.3 Assessment of factorability

The correlation matrix was highly suitable for factor analysis, with a Kaiser–Meyer–Olkin measure of sampling adequacy of 0.975 (individual item KMOs ranged from 0.945 to 0.986) and a significant Bartlett’s test of sphericity, χ^2^(276) = 9564.24, p < 0.001, confirming sufficient common variance for factor extraction.

### 4.4 Second-order confirmatory factor analysis

**Figure 1:**
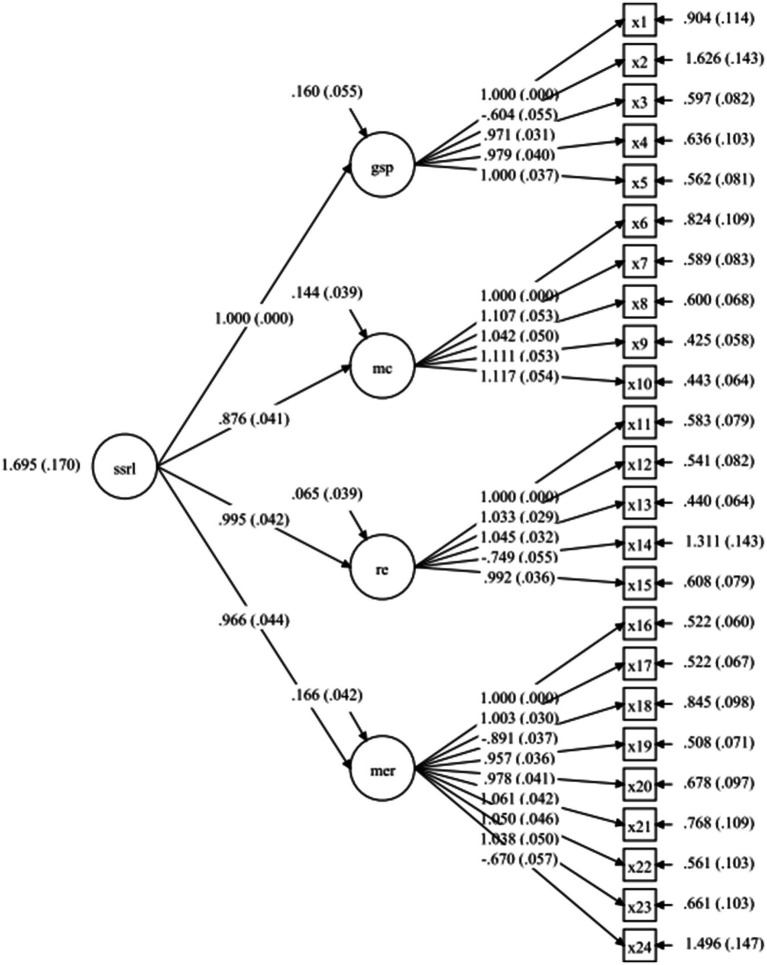
Second-order confirmatory factor analysis model of SSRL.

Given the departure from multivariate normality, MLR estimation was employed to provide robust parameter estimates and fit indices (see Figure 1). The second-order CFA demonstrated good fit (Table 2): χ^2^(248) = 484.34, *p* < 0.001; CFI = 0.951; TLI = 0.946; RMSEA = 0.050 (90% CI [0.044, 0.057], p(RMSEA ≤ 0.05) = 0.470); SRMR = 0.038. Standardized loadings from the four first-order dimensions onto the higher-order SSRL factor were all strong and significant—0.956 for Shared Goal Setting and Planning, 0.949 for Shared Monitoring and Control, 0.981 for Shared Reflection and Evaluation, and 0.951 for Shared Motivational and Emotional Regulation—thereby confirming the hypothesized hierarchical model, with all four first-order dimensions showing strong convergence onto the higher-order SSRL factor. These high standardized loadings indicate that the dimensions are closely related and together represent a coherent higher-order construct.

**Table 2:** Fit indices for second-order CFA model of SSRL.

Fit index	Value	Criterion
χ^2^ (df)	484.34 (248), *p* < 0.001	—
CFI	0.951	≥ 0.90
TLI	0.946	≥ 0.90
RMSEA (90% CI)	0.050 (0.044, 0.057)	≤ 0.08
SRMR	0.038	≤ 0.08

## 5 Discussion

The present study sought to address the longstanding need for a psychometrically rigorous, multidimensional instrument to assess socially shared regulated learning (SSRL). Consistent with the hierarchical framework proposed by Hadwin and Oshige (2011), our second-order CFA confirmed four first-order dimensions—Shared Goal Setting and Planning, Shared Monitoring and Control, Shared Reflection and Evaluation, and Shared Motivational and Emotional Regulation—loading strongly onto a higher-order SSRL factor. Fit indices (CFI = 0.951; RMSEA = 0.050; SRMR = 0.038) and reliability coefficients (*ω*’s > 0.82) all met or exceeded conventional thresholds (Panadero, 2017; Zimmerman, 2000), indicating that the instrument captures both the cognitive–metacognitive and the socio-emotional facets of collaborative regulation. These findings extend prior work on individual SRL (Pintrich, 2000; Zimmerman and Kitsantas, 1997) by empirically validating the shift from intra- to inter-personal regulatory processes in group contexts. In particular, the strong loadings for Shared Motivational and Emotional Regulation underscore the critical role of socio-emotional support and collective motivation in sustaining effective group engagement—an area often underrepresented in earlier SSRL measures (Volet et al., 2009). Moreover, the addition of reverse-scored items enhanced discriminability without compromising scale coherence, suggesting that future SSRL assessments should balance positively and negatively keyed items to mitigate response biases. Collectively, these psychometric results establish a solid basis for applied use.

Beyond measurement, the validated SSRL instrument offers a practical formative diagnostic for team-based and inquiry-oriented courses in higher education. Used formatively, it enables early detection of regulatory bottlenecks, targeted scaffolds at key milestones, and ongoing monitoring within peer-assessment workflows and learning-analytics dashboards. Because the items index general collaborative regulation behaviors rather than culture-specific practices, the instrument is likely portable across higher education systems.

Despite these strengths, the study’s reliance on convenience sampling within Chinese public universities and cross-sectional survey data may limit generalizability. Subsequent research should examine the instrument’s predictive validity—linking SSRL scores to objective learning outcomes—and test its applicability across diverse cultural and disciplinary settings. Longitudinal studies would also illuminate how SSRL processes evolve over time and in response to targeted pedagogical interventions. Additionally, although the MLR estimator was employed to address the significant departure from multivariate normality, this violation of the distributional assumption may still have introduced minor biases in parameter estimates or affected statistical power. Future research could examine whether similar results are obtained using alternative robust estimators or bootstrapping techniques.

## 6 Conclusion

In conclusion, this research fills a critical methodological gap by developing and validating a comprehensive 24-item SSRL questionnaire grounded in contemporary theory. The demonstrated construct validity, excellent internal consistency, and clearly articulated hierarchical factor structure provide strong evidence that the instrument effectively measures both the cognitive–metacognitive and socio-emotional dimensions of socially shared regulation. By capturing how groups co-construct goals, monitor progress, engage in reflective evaluation, and sustain motivational support, this tool not only advances SSRL theory but also offers a precise lens through which to observe and quantify dynamic team-based learning processes.

Practically, educators and instructional designers can leverage this 24-item SSRL questionnaire—explicitly designed for large-scale administration with a clear seven-point Likert format and streamlined structure—to diagnose specific regulatory strengths and weaknesses within student teams, tailor interventions such as structured goal-setting workshops or affective support prompts, and monitor the impact of pedagogical changes over time. Its robust psychometric properties and efficient deployment—across face-to-face seminars, online collaborative platforms, and professional development settings in both STEM and humanities—allow researchers to administer it to hundreds or even thousands of participants without sacrificing measurement precision. Consequently, teams can be reliably benchmarked across cohorts, institutions, and disciplines, SSRL assessment can be integrated into longitudinal or multi-site evaluations, and consistent, detailed scores on goal setting, monitoring, reflection, and motivational support fulfill the need for a psychometrically sound, easily scalable measure of socially shared regulation in any context requiring extensive sampling.

## Data Availability

The original contributions presented in the study are included in the article/Supplementary material, further inquiries can be directed to the corresponding author.

## Supplementary material

SUPPLEMENTARY MATERIAL 1 Full SSRL questionnaire (PDF).

SUPPLEMENTARY MATERIAL 2 TAP transcripts (Excel).

SUPPLEMENTARY MATERIAL 3 SSRL questionnaire raw data (Excel).
