# Supplementary material for: Development and validation of the socially shared regulated learning questionnaire: insights from a second-order confirmatory factor analysis
Source: Front Psychol. 2025 Sep 8;16:1635325. doi: 10.3389/fpsyg.2025.1635325 (PMC12450926; doi:10.3389/fpsyg.2025.1635325)
Supplement: SUPPLEMENTARY MATERIAL 1 — Full SSRL questionnaire (PDF). [file Data_Sheet_1.PDF]

Socially Shared Regulated Learning

Name: ________________________ **Gender:** **Male** ☐ **Female** ☐

Student ID: ________________________ **Age**__________________________

Academic Year: **Freshman** ☐ **Sophomore** ☐ **Junior** ☐ **Senior** ☐

Notes: Please read the following questions carefully and evaluate accurately how often you engage in the following socially shared regulation of learning practices during collaborative learning activities. These questions are designed to assess your perception of frequency in these practices, so there are no right or wrong answers. The data collected from this questionnaire will be used exclusively for research purposes, and your personal sensitive information will be kept confidential. You may proceed with completing the questionnaire if you consent.

Please use the following scales to answer these questions accordingly. Please choose the number accurately representing your abilities.

1-Never, 2-Very rarely, 3-Rarely, 4-Occasionally, 5-Sometimes, 6-Often, 7-Always

Items		1	2	3	4	5	6	7
1.	I collaborate with my group to set clear learning goals for our tasks.							
2.	I avoid discussing with group members when the goals set by our group deviate from the team's direction.							
3.	When our group fails to meet learning objectives, I actively collaborate with other members to adjust our learning strategies.							
4.	I ensure that everyone's ideas are considered when we establish our goals.							
5.	I co-construct standards with my group to measure our collective success.							
6.	I regularly check on my group members' progress during our activities.							
7.	I provide constructive feedback to my group members to help improve our work.							
8.	I keep my group members informed about any challenges I encounter.							

9.	I evaluate our group’s performance to ensure we stay on track.							
10.	I openly discuss any deviations from our planned approach with my group.							
11.	After completing a task, I reflect with my group on what we learned.							
12.	I discuss with my group about what strategies worked well and what didn’t.							
13.	I analyze the outcomes of our collaborative efforts together with my group.							
14.	I seldom reflect with my group on how we can improve our collaboration in future tasks.							
15.	I share personal insights to enhance our group’s understanding.							
16.	I adapt my behaviors based on feedback from my group members.							
17.	I influence my group members’ learning strategies in a positive way.							
18.	I focus more on achieving my own goals in our group.							
19.	I dynamically adjust our plans with my group in response to new information.							
20.	I enjoy exchanging ideas with my group about how we approach tasks.							
21.	I trust and respect my group members’ contributions.							
22.	I am actively involved in the collaborative decision-making process in our group.							
23.	I support the personal growth of each member within our group setting.							
24.	If there are disagreements within the group, I tend to avoid interacting with the members.							
